# Systemic Inflammatory Indices (SII and SIRI) in 30-Day Mortality Risk Stratification for Community-Acquired Pneumonia: A Study Alongside CURB-65 and PSI

**DOI:** 10.3390/pathogens14121235

**Published:** 2025-12-03

**Authors:** Orkun Eray Terzi, Gülgün Çetintaş Afşar, Nazlı Çetin, Seyhan Dülger

**Affiliations:** 1 Department of Pulmonology, Bursa Yuksek Ihtisas Training and Research Hospital, 16310 Bursa, Türkiye; gulgun.cetintasafsar@sbu.edu.tr (G.Ç.A.); seyhan.dulger@sbu.edu.tr (S.D.); 2 Department of Pulmonology, Afyonkarahisar State Hospital, 03100 Afyonkarahisar, Türkiye; nazlicetinbeyaz@gmail.com

**Keywords:** community-acquired pneumonia, systemic inflammation, SII, SIRI, CURB-65, PSI, prognostic biomarkers, mortality prediction

## Abstract

Community-acquired pneumonia (CAP) remains a leading cause of morbidity and mortality worldwide, underscoring the need for accessible and cost-effective biomarkers to support early risk assessment. This retrospective study investigated the prognostic performance of two systemic inflammatory indices—the Systemic Immune-Inflammation Index (SII) and the Systemic Inflammation Response Index (SIRI)—in 240 adults hospitalized with CAP between January and December 2024. The primary outcome was 30-day all-cause mortality. Logistic regression and receiver operating characteristic (ROC) analyses were applied to compare these indices with established severity scores, CURB-65 and the Pneumonia Severity Index (PSI). Thirty-day mortality occurred in 15.4% of patients. Non-survivors exhibited significantly higher SII values (*p* = 0.043) and a trend toward increased SIRI levels (*p* = 0.072). Both indices showed weak but statistically significant positive correlations with conventional inflammatory markers such as C-reactive protein and procalcitonin. While CURB-65 and PSI retained superior discriminative ability, SII and SIRI provided only modest prognostic information and did not significantly improve mortality prediction beyond these scores. These findings indicate that simple, hematology-based indices reflecting systemic inflammation may offer limited but potentially clinically relevant adjunctive information when integrated with traditional clinical scoring systems.

## 1. Introduction

Community-acquired pneumonia (CAP) remains a leading cause of infectious morbidity and mortality worldwide [1]. The clinical course becomes increasingly heterogeneous with advancing age and the presence of comorbidities, making mortality prediction particularly challenging. Early identification of high-risk patients is therefore essential to optimize treatment intensity, allocate healthcare resources effectively, and guide appropriate antibiotic strategies [2].

Clinical scoring systems such as the CURB-65 and the Pneumonia Severity Index (PSI) were developed to stratify mortality risk in a practical manner and are now widely incorporated into routine clinical decision making [3,4]. However, these models are primarily based on demographic characteristics, vital parameters, and comorbid conditions, and therefore fail to capture the biological severity of systemic inflammation.

In recent years, hematological indices derived from complete blood count parameters have been introduced to allow a more objective assessment of the inflammatory response. Among these, the Systemic Immune-Inflammation Index (SII) and the Systemic Inflammation Response Index (SIRI) have gained particular prominence. The SII (neutrophil × platelet/lymphocyte) was first proposed by Hu et al. in patients with hepatocellular carcinoma, whereas the SIRI (neutrophil × monocyte/lymphocyte) was subsequently defined by Qi et al. in a pancreatic cancer cohort [5,6]. Both indices combine pro-inflammatory (neutrophils, monocytes, platelets) and immunoregulatory (lymphocytes) components into a single numerical parameter that quantifies the host’s systemic inflammatory balance. Activation of neutrophils and platelets during the early phase of systemic inflammation, followed by lymphocyte suppression in later stages, provides the biological rationale for these indices. Accordingly, it has been proposed that SII and SIRI may provide additional prognostic power to conventional clinical scoring systems by reflecting the systemic impact of infection. Nevertheless, this hypothesis has not yet been adequately validated in acute infectious conditions particularly in CAP cohorts using multivariable frameworks.

In the current literature, evidence regarding the prognostic value of SII and SIRI in CAP remains limited and methodologically heterogeneous. Most existing studies have relied on univariate analyses or limited comparisons based solely on receiver operating characteristic (ROC) curves, whereas investigations evaluating their independent prognostic contribution within multivariable models that include established clinical severity scores remain scarce [7,8,9].

Therefore, the primary objective of this study was to assess the prognostic performance of the systemic inflammation indices SII and SIRI for predicting 30-day all-cause mortality in adults hospitalized with CAP, both as stand-alone markers and in combination with the established clinical severity scores CURB-65 and PSI, using logistic regression and ROC–DeLong analyses. As a secondary objective, we investigated the associations between SII/SIRI, clinical severity scores (CURB-65 and PSI), and biochemical inflammatory biomarkers (C-reactive protein and procalcitonin) to provide a more comprehensive understanding of the prognostic utility of accessible hematologic biomarkers in CAP risk stratification.

## 2. Materials and Methods

### 2.1. Study Design and Ethical Approval

This retrospective, single-center study was conducted at the University of Health Sciences, Bursa Yuksek Ihtisas Training and Research Hospital (Bursa, Türkiye). The study protocol was reviewed and approved by the Ethics Committee of the University of Health Sciences, Bursa Yuksek Ihtisas Training and Research Hospital (Approval No: 2024-TBEK 2025/06-05, dated 18 June 2025) and carried out in accordance with the ethical principles outlined in the Declaration of Helsinki. The requirement for informed consent was waived due to the retrospective design of the study.

### 2.2. Study Population

**Figure 1:**
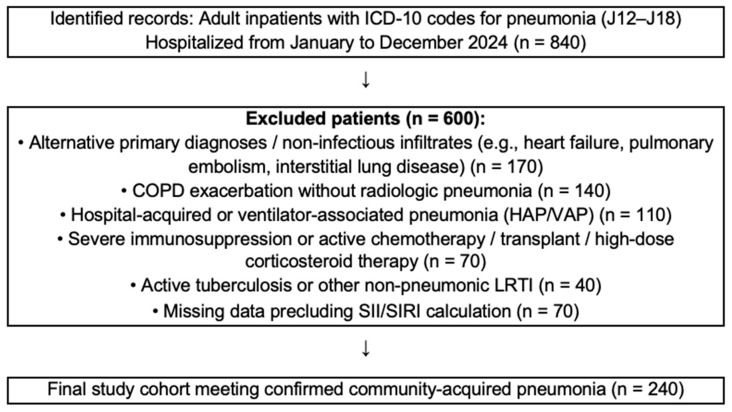
Study flow diagram illustrating patient inclusion and exclusion. Numbers are mutually exclusive; each excluded patient was counted once according to the primary exclusion reason.

Adult patients (≥18 years) who were hospitalized with a diagnosis of community-acquired pneumonia (CAP)—ICD-10 codes J12–J18—between 1 January 2024 and 31 December 2024, were retrospectively identified from the hospital information system. A total of 840 patients were screened, of whom 240 met the final inclusion criteria and constituted the final study cohort (Figure 1). Among the excluded patients, 70 out of 840 (8.3%) were removed because one or more baseline laboratory parameters required to calculate SII or SIRI (full blood count components and/or C-reactive protein or procalcitonin) were missing. In the final analytic cohort, baseline data for SII, SIRI, CURB-65, PSI, key covariates, and 30-day all-cause mortality status were complete. Community-acquired pneumonia was diagnosed according to guideline-based criteria from the 2021 Turkish Thoracic Society CAP guideline, requiring a new pulmonary infiltrate on chest radiograph or CT compatible with infection, together with compatible respiratory symptoms (e.g., new or worsening cough, sputum production, dyspnea, pleuritic chest pain) and at least one systemic sign such as fever, hypothermia, leukocytosis or leukopenia.

Exclusion criteria were as follows:

(1) Age < 18 years;
(2) Hospital-acquired or ventilator-associated pneumonia (HAP/VAP);
(3) Severe immunosuppression or active chemotherapy, solid organ transplantation, or chronic corticosteroid therapy (>20 mg/day for ≥2 weeks);
(4) Active pulmonary tuberculosis or other non-pneumonic lower respiratory tract infection;
(5) Chronic obstructive pulmonary disease (COPD) exacerbation without radiologic pneumonia;
(6) Alternative primary diagnoses or non-infectious infiltrates (e.g., heart failure, pulmonary embolism, interstitial lung disease);
(7) Missing data precluding the calculation of SII/SIRI.

### 2.3. Data Collection

The following variables were extracted from the electronic hospital records at the time of admission: demographic data (age, sex, and smoking history); clinical characteristics, including comorbidities such as COPD, diabetes mellitus, hypertension, coronary artery disease, cerebrovascular disease, and malignancy; and recent antibiotic use or hospitalization within the previous 90 days. Laboratory parameters included total leukocyte, neutrophil, lymphocyte, monocyte, and platelet counts, as well as CRP and PCT levels. Disease severity was assessed using the CURB-65 and PSI scores.

Systemic inflammatory indices were calculated as follows:

Systemic Immune-Inflammation Index (SII) = platelet × (neutrophil/lymphocyte)

Systemic Inflammation Response Index (SIRI) = (neutrophil × monocyte)/lymphocyte

All patients were managed according to institutional practice based on the 2021 Turkish Thoracic Society guideline for the management of community-acquired pneumonia. Empirical antibiotic regimens were selected by the treating physicians in line with this guideline, according to disease severity and individual risk factors. Complete blood counts and routine biochemistry used to calculate SII and SIRI were obtained at hospital admission, before or within the first 24 h after the initiation of guideline-based empirical antibiotic therapy.

### 2.4. Outcomes

The primary outcome of the study was 30-day all-cause mortality among patients hospitalized with community-acquired pneumonia. Secondary analyses examined the relationships between systemic inflammatory indices (SII and SIRI) and conventional inflammatory biomarkers, including CRP and PCT. Additionally, correlations between these indices and established clinical severity scores (CURB-65 and PSI) were assessed. Finally, the incremental discriminative value of incorporating SII and SIRI into existing clinical models (CURB-65 and PSI) for predicting 30-day mortality was evaluated.

### 2.5. Statistical Analysis

All statistical analyses were performed using IBM SPSS Statistics version 29.0 (IBM Corp., Armonk, NY, USA) and MedCalc Statistical Software version 22.0 (MedCalc Software Ltd., Ostend, Belgium). The normality of continuous variables was assessed using the Kolmogorov–Smirnov test. Distributional assumptions were also inspected via histograms and Q-Q plots. Continuous variables were summarized as mean ± standard deviation (SD) or median [interquartile range (IQR)], while categorical variables were expressed as counts and percentages. Between-group comparisons were conducted using Student’s *t*-test or Mann–Whitney U test for continuous data, and the chi-square (χ^2^) test or Fisher’s exact test for categorical variables, as appropriate. Correlations between systemic inflammatory indices and clinical or laboratory parameters were analyzed using Spearman’s rank correlation coefficient (ρ). Independent predictors of 30-day mortality were identified by binary logistic regression analysis, performed separately for clinical scores (CURB-65, PSI) and systemic inflammatory indices (SII, SIRI). Combined models incorporating both clinical scores and inflammatory indices were subsequently constructed to evaluate their incremental prognostic value. Model calibration was assessed using the Hosmer–Lemeshow goodness-of-fit test, and no evidence of multicollinearity was observed among covariates (all VIF < 2). The discriminative performance of individual and combined models was evaluated using receiver operating characteristic (ROC) curve analysis. ROC curves were constructed to assess the discriminative performance of SII, SIRI, CRP, procalcitonin, CURB-65, PSI, and the combined models for 30-day mortality. For each marker, the optimal cut-off value was determined by maximizing the Youden index (J = sensitivity + specificity − 1), and the corresponding sensitivity and specificity estimates at this cut-off are reported. Differences between areas under the curve (AUCs) were compared using the DeLong method, with 95% confidence intervals (CIs) calculated for all estimates. All statistical tests were two-tailed, and a *p*-value < 0.05 was considered statistically significant.

## 3. Result

### 3.1. Demographic, Clinical, and Laboratory Characteristics

A total of 240 patients hospitalized with community-acquired pneumonia were analyzed. The median age was 75.5 years (range, 21–95), and 60.8% were male. Thirty-day all-cause mortality occurred in 37 patients (15.4%). The study population was divided into two groups based on 30-day survival status: survivors and non-survivors. Non-survivors were older than survivors (76.5 ± 11.9 vs. 75.0 years), although this difference was not statistically significant (*p* = 0.104). Male sex was more frequent among non-survivors than survivors (73.0% vs. 59.3%, *p* = 0.07). Recent hospitalization within 90 days and prior antibiotic exposure were both significantly associated with 30-day mortality (*p* = 0.015 and *p* = 0.026, respectively). Overall comorbidity burden was higher among non-survivors (*p* = 0.082); however, malignancy was the only comorbidity significantly associated with short-term death (*p* < 0.001). Other comorbidities, including diabetes mellitus, hypertension, coronary artery disease, cerebrovascular disease, and COPD, did not differ significantly between survivors and non-survivors. Disease severity indices were also markedly higher among non-survivors: PSI (143.84 ± 37.50 vs. 107.38 ± 31.50, *p* < 0.001) and CURB-65 (≥3 in 35.1% vs. 9.9%, *p* < 0.001).

In laboratory analyses, non-survivors exhibited significantly lower lymphocyte counts (*p* = 0.013) and a higher systemic inflammatory burden, reflected by elevated SII (*p* = 0.043) and PCT levels (*p* = 0.014). No significant differences were observed in other hematologic or biochemical parameters, including total leukocyte, neutrophil, or CRP levels. Detailed demographic, clinical, and laboratory characteristics according to 30-day survival status are summarized in Table 1. The overall pattern of these associations remained largely unchanged after adjustment for age, sex, and malignancy; adjusted *p*-values are presented in Table 1.

### 3.2. Baseline Correlation Analysis of Inflammatory and Clinical Parameters

At admission, systemic inflammatory indices showed a strong internal correlation and only weak-to-modest associations with laboratory biomarkers (Table 2). SII and SIRI were highly correlated with each other (Table 2). SII and SIRI were highly correlated with each other (ρ = 0.711, *p* < 0.001). Both indices showed moderate correlations with CRP (SII: ρ = 0.275, *p* < 0.001; SIRI: ρ = 0.347, *p* < 0.001) and PCT (SII: ρ = 0.209, *p* = 0.001; SIRI: ρ = 0.278, *p* < 0.001). In addition, SII and SIRI demonstrated weak but statistically significant correlations with CURB-65 (ρ = 0.216 and ρ = 0.183, respectively) and PSI scores (ρ = 0.199 and ρ = 0.132, respectively).

Correlations among dynamic changes (Δ) in inflammatory biomarkers during hospitalization were then examined. ΔSII and ΔSIRI were significantly correlated (ρ = 0.566, *p* < 0.001). ΔProcalcitonin showed a moderate correlation with ΔCRP (ρ = 0.455, *p* < 0.001) and a weak correlation with ΔSIRI (ρ = 0.224, *p* = 0.023). No significant correlations were observed between ΔSII and ΔCRP (*p* = 0.772) or between ΔSII and ΔProcalcitonin (*p* = 0.106).

### 3.3. Logistic Regression Analysis for Predictors of 30-Day Mortality

Binary logistic regression analyses were conducted to evaluate the independent associations between clinical severity scores, systemic inflammatory indices, conventional inflammatory biomarkers, and 30-day mortality. In univariate models, higher CURB-65 and PSI scores and higher ln-SII and ln-SIRI values were significantly associated with increased odds of 30-day mortality (all *p* < 0.05), whereas CRP and procalcitonin were not significant predictors (*p* = 0.120 and *p* = 0.166, respectively) (Table 3).

Subsequently, bivariate models were constructed by adding SII or SIRI to each clinical score to assess their incremental prognostic value. In these combined models, CURB-65 and PSI remained significantly associated with 30-day mortality (*p* < 0.001 for all), while SII and SIRI did not retain statistical significance after adjustment (*p* ≈ 0.05–0.15) (Table 3). Regression coefficients, standard errors, odds ratios (ORs), and 95% confidence intervals (CIs) for all models are presented in Table 3. No evidence of multicollinearity was observed among covariates included in the combined models, and all models demonstrated acceptable goodness-of-fit (Hosmer–Lemeshow test, *p* > 0.05).

To complement the regression results, ROC analyses were performed to quantify the discriminative performance of individual and combined models for 30-day mortality.

### 3.4. Discriminative Performance of Predictive Models (ROC Analysis)

ROC analysis was performed to evaluate the discriminative performance of clinical scores, systemic inflammatory indices, and inflammatory biomarkers for predicting 30-day mortality (Table 4). CRP showed poor discrimination (AUC 0.569, 95% CI 0.467–0.671, *p* = 0.187), whereas procalcitonin demonstrated only modest discriminatory ability (AUC 0.627, 95% CI 0.531–0.723, *p* = 0.010). The systemic inflammatory indices also showed modest but consistent discriminative ability, with an AUC of 0.605 (95% CI 0.540–0.667, *p* = 0.047) for SII and 0.609 (95% CI 0.544–0.672, *p* = 0.042) for SIRI. Among the clinical scoring systems, CURB-65 yielded an AUC of 0.684 (95% CI 0.621–0.742), whereas PSI achieved 0.768 (95% CI 0.709–0.820), indicating superior discriminative performance compared with SII, SIRI, CRP, and procalcitonin. When either inflammatory index was incorporated into these baseline models, the combined AUCs were 0.723 (95% CI 0.662–0.779) for CURB-65 + SII, 0.727 (95% CI 0.665–0.782) for CURB-65 + SIRI, 0.774 (95% CI 0.715–0.825) for PSI + SII, and 0.776 (95% CI 0.718–0.827) for PSI + SIRI. Optimal cut-off values, together with corresponding sensitivity and specificity, are summarized in Table 4. 

Pairwise AUC comparisons based on the DeLong method are presented in Table 4, together with the corresponding ΔAUC values and 95% confidence intervals. None of the pairwise AUC differences between the combined models and their respective baseline clinical scores reached statistical significance. Model calibration, evaluated using the Hosmer–Lemeshow test, was acceptable for all models. Although the addition of SII or SIRI to CURB-65 or PSI resulted in slightly higher AUC values, these changes did not reach statistical significance.

ROC analysis demonstrated a modest, incremental improvement in discriminative performance when SII and SIRI were added to the baseline clinical models, although these changes did not reach statistical significance.

## 4. Discussion

In this study, we investigated the prognostic value of systemic inflammation indices (SII and SIRI) for short-term mortality in hospitalized patients with CAP. Our findings indicate that SII was significantly associated with 30-day mortality, whereas SIRI demonstrated only a borderline association. The established clinical severity scores, CURB-65 and PSI, remained stronger predictors of mortality, and adding SII or SIRI to these models resulted in only limited improvement in discriminative performance. These results suggest that systemic inflammatory indices may play an independent but secondary role in CAP prognosis.

Correlation analyses revealed weak but statistically significant associations between SII and SIRI and biochemical inflammatory markers (CRP and procalcitonin) yet only weak correlations with clinical severity scores. This discrepancy highlights that biochemical inflammatory markers do not fully capture the clinical manifestations of disease severity. These findings suggest that the clinical course of CAP is shaped by complex determinants—such as host-specific immune dynamics and systemic physiological reserve—extending beyond the information provided by conventional biomarkers.

A strong correlation between SII and SIRI further supports the notion that both indices primarily reflect a neutrophil-dominant systemic inflammatory response. However, because SII is also influenced by platelet and lymphocyte counts, it may capture not only cellular activation but also the interplay between immune regulation and hemostatic balance. This broader biological scope could partially explain the slightly superior prognostic performance of SII compared with SIRI in our cohort.

In this study, we examined the baseline clinical and laboratory characteristics of patients diagnosed with CAP. The data obtained reflect the demographic composition and initial clinical profile of the study population at hospital admission. Both widely used pneumonia severity scores, CURB-65 and PSI, incorporate age as a central component. Specifically, the CURB-65 score directly includes the criterion of age ≥ 65 years, whereas the PSI assigns the highest weighting to age among its variables [3,4]. These scoring principles underscore that advanced age remains a critical determinant of mortality risk in CAP. A meta-analysis demonstrated that the incidence of CAP is higher in men and increases progressively with age [10]. Historically, male sex has been identified as a factor associated with higher mortality risk [11,12]; however, this association has not been consistently reproduced across studies [13,14]. In our cohort, elderly patients constituted the majority of cases (median age, 75.5 years; range, 21–95). This distribution aligns with previously reported CAP populations and supports the notion that age is a key determinant of disease trajectory. Although non-survivors were older on average than survivors (76.5 ± 11.9 vs. 75 years, *p* = 0.104), the difference was not statistically significant. The trend, however, remains clinically relevant given the established impact of advanced age on CAP prognosis. Male sex accounted for 60.8% overall and 73% among non-survivors (*p* = 0.07), a pattern consistent with reports of higher incidence and worse outcomes in men, though our study may have been underpowered to detect a significant difference.

Recent antibiotic use and hospitalization within the previous 90 days are important factors that may influence the clinical course of CAP. In our study, both variables were found to be significantly associated with 30-day mortality. The existing literature has long established that recent hospitalization and antibiotic exposure increase the risk of infection with drug-resistant pathogens [15]. In a recent review, these clinical variables were also highlighted as factors associated with poor prognosis in severe community-acquired pneumonia [16]. Our findings suggest that these factors may influence not only microbiological outcomes related to resistant pathogens but also overall mortality risk.

The burden of comorbidities is among the most important patient-related factors that determine prognosis in CAP. Previous studies consistently report that the presence of one or more comorbidities significantly increases short-term mortality in CAP [17]. However, it has also been reported that, particularly in the very elderly population (≥80 years), having fewer comorbidities may paradoxically serve as an independent risk factor for mortality [18]. The prognostic impact of comorbidities is not homogeneous. It has been reported that diabetes mellitus, chronic obstructive pulmonary disease (COPD), and smoking history do not significantly contribute to mortality risk, whereas the presence of cancer, cerebrovascular disease, and ischemic heart disease markedly increases the risk of death [17]. Similarly, another study identified the presence of malignancy and COPD as the only independent predictors of mortality [19]. In our study, the overall presence of comorbidities was associated with a trend toward higher mortality, although this did not reach statistical significance (*p* = 0.082). However, subgroup analyses demonstrated that the presence of malignancy was strongly associated with short-term mortality (*p* < 0.001). In contrast, no significant differences were observed for diabetes mellitus, hypertension, coronary artery disease, cerebrovascular disease, or COPD. These findings may be related to the immunosuppressive and inflammation-modulating effects of malignancy on the host immune response and systemic inflammatory balance. The prognostic impact of other comorbidities may have been partly obscured by factors such as age, functional capacity, and nutritional status.

In the literature, elevated neutrophil counts and reduced lymphocyte levels in patients with CAP have been shown to be closely associated with increased mortality. This imbalance is thought to reflect a transition of the host immune response from hyperinflammation toward immunosuppression [20,21,22]. Consistent with previous reports, our study also found significantly lower lymphocyte counts in the non-survivor group (*p* = 0.013). In contrast, neutrophil and total leukocyte levels did not differ significantly between groups. This finding suggests that the immune response associated with mortality may be determined not by the magnitude but by the qualitative composition of inflammation—particularly lymphopenia. A reduction in lymphocyte count may limit the host’s ability to cope with infection, serving as an indicator of both systemic stress response and immune dysfunction.

CRP is considered a marker of the host’s response to inflammatory cytokines associated with monocyte and macrophage activation, and its expression increases under inflammatory conditions. In the literature, inflammatory biomarkers have consistently been reported to differ significantly between survivors and non-survivors, with markedly higher CRP levels observed in patients with fatal outcomes [23]. In our study, CRP levels did not differ significantly between the groups (*p* = 0.157); however, higher mean values observed among non-survivors were consistent with the trend reported in prior studies. In contrast, PCT levels were significantly higher in the non-survivor group (*p* = 0.014). This finding aligns with meta-analytic evidence, as recent studies have demonstrated that elevated PCT levels are strongly associated with increased mortality in patients with CAP [24].

Elevated SII and SIRI levels have been reported to be significantly associated with mortality across various disease settings, including sepsis, chronic kidney disease, cardiovascular disorders, and malignancies [25,26,27]. These findings suggest that both indices reflect not only local inflammation but also the host-related systemic inflammatory burden and immune dysregulation. Therefore, it is hypothesized that similar mechanisms may also apply to acute infections, and the number of studies investigating the prognostic value of SII and SIRI in CAP has been steadily increasing. In a cohort of 345 patients with CAP, SII was found to have significant prognostic value for predicting 28-day mortality [8]. In another study evaluating patients with severe pneumonia, the combination of SII and the nutritional status parameter PNI (SII–PNI score) was shown to strongly predict 28-day mortality [9]. In a recent study involving 207 patients with CAP, several inflammatory indices were compared. Both SII and SIRI were found to be significantly associated with mortality [7]. Consistent with these findings, our study also demonstrated significantly higher SII values in the non-survivor group (*p* = 0.043), whereas SIRI showed only a borderline association with mortality (*p* = 0.072). This suggests that, in the acute course of pneumonia, monocyte-mediated inflammation may play a secondary role, whereas neutrophil and platelet activation accompanied by lymphopenia appears to be more decisive for mortality. Therefore, SII may serve as a more sensitive and early-responding biomarker for predicting mortality in CAP, whereas SIRI may provide additional information in conditions accompanied by chronic or systemic inflammation.

Previous studies examining the relationship between systemic inflammatory indices and conventional biomarkers have shown that SII and SIRI are moderately correlated with CRP and PCT. A recent study reported that, in patients with CAP, CRP levels showed significant positive correlations with leukocyte count, neutrophil count, SII, and SIRI; however, the strength of these associations remained moderate. Another study highlighted that the correlation between SII and CRP was limited, suggesting that these two parameters may reflect different aspects of the inflammatory response [7,8]. Our findings are consistent with these reports: SII and SIRI showed weak but statistically significant correlations with CRP and PCT, but only weak correlations with clinical severity scores (PSI and CURB-65). These results suggest that hematological inflammation indices and biochemical markers reflect the same inflammatory process from different perspectives and may therefore serve as complementary indicators.

Previous studies have primarily compared SII and SIRI with clinical severity scores at the ROC-curve level; however, evidence assessing their incremental prognostic contribution within multivariable models remains limited. In the literature, SII has been shown to possess significant prognostic value for mortality and to achieve improved predictive performance when combined with additional risk dimensions such as nutritional status (e.g., PNI) [8,9]. Our study addressed this gap by incorporating SII and SIRI into the CURB-65 and PSI models and testing their performance using both logistic regression and ROC–DeLong analyses. The findings indicated that the addition of SII to these established clinical scores did not result in a statistically significant improvement, whereas SIRI provided only a borderline contribution (*p* = 0.052–0.065). A similar trend was observed in the ROC analysis, where the addition of SII or SIRI yielded only small ΔAUC values (0.039–0.043) and failed to reach statistical significance (DeLong *p* > 0.05). Within the same ROC framework, CRP and procalcitonin also showed only limited discriminatory ability, with AUC values lower than or comparable to those of SII and SIRI and clearly inferior to CURB-65 and PSI, indicating that both hematology-based and biochemical inflammatory markers share similar limitations when compared with established clinical scores.

These findings suggest that hematologic indices may serve as complementary rather than substitutive parameters to established clinical scoring systems in risk stratification. To better demonstrate clinically meaningful incremental value, future studies could incorporate advanced statistical approaches beyond traditional AUC comparison, including reclassification metrics (NRI/IDI), calibration analyses (Hosmer–Lemeshow test and calibration curves), and decision-curve analysis. Moreover, dynamic models integrating external validation, time-dependent analyses, and serial measurements (ΔSII/ΔSIRI) may more accurately capture the evolving nature of the inflammatory response. Nonetheless, current evidence consistently indicates that systemic inflammatory indices provide only a limited additional contribution to short-term mortality prediction in CAP.

This study has several limitations. First, its single-center and retrospective design carries an inherent risk of selection bias and incomplete data capture. Furthermore, inflammatory biomarkers were measured only at hospital admission; thus, temporal changes in inflammatory parameters (ΔSII, ΔSIRI, ΔCRP, Δprocalcitonin) could be assessed only in a limited subset of patients. As a result, the dynamic course of the systemic inflammatory response and its impact on mortality could not be fully elucidated. Third, we did not systematically record all concomitant medications administered around the time of admission (e.g., short courses of systemic corticosteroids or other agents that may affect leukocyte counts); therefore, a residual impact of such treatments on SII and SIRI values cannot be completely excluded. In addition, heterogeneity in comorbidity profiles, causative pathogens, antibiotic susceptibility patterns, and treatment strategies may limit the generalizability of the findings. The cut-off values used in the analyses may also vary across populations, underscoring the need for standardization before clinical implementation of SII and SIRI. Finally, since the study cohort primarily consisted of elderly patients with multiple comorbidities, extrapolation of the results to younger or community-based populations should be made with caution.

## 5. Conclusions

Systemic inflammatory indices such as SII and SIRI provide accessible, hematology-based reflections of the immune–inflammatory imbalance underlying community-acquired pneumonia. In this single-center cohort, higher SII and SIRI values at admission were associated with 30-day all-cause mortality; however, these indices demonstrated only modest discriminative ability and did not outperform established clinical scores. CURB-65 and, in particular, the Pneumonia Severity Index remained the strongest predictors of short-term mortality. When SII or SIRI was added to CURB-65 or PSI, the resulting combined models yielded only small, non-significant increases in AUC, and the indices did not retain independent significance in multivariable logistic regression. Conventional biochemical markers such as CRP and procalcitonin also showed limited discriminatory performance and were not clearly superior to SII or SIRI.

Taken together, these findings indicate that SII and SIRI should not be used as stand-alone risk stratification tools in CAP but may be considered as simple, low-cost adjunctive markers that provide additional biological context on host inflammatory response when interpreted alongside established clinical severity scores. Larger, prospective, multicenter and longitudinal studies—including dynamic assessments of biomarker trajectories—are warranted to validate these observations and to clarify whether integrating hematology-derived inflammatory indices into composite risk-based triage or antibiotic stewardship algorithms can offer clinically meaningful improvements in early identification of high-risk patients and optimization of therapeutic decision making

## Figures and Tables

**Table 1:** Baseline demographic, clinical, and laboratory characteristics of patients according to 30-day survival status.

Variable	Overall (*n* = 240)	Survivors (*n* = 203)	Non-Survivors (*n* = 37)	*p*-Value (Unadjusted)	*p* (Adjusted) *
Age	75.50 (21–95)	72.02 ± 14.41	76.54 ± 11.94	0.104	-
Male sex, n (%)	146 (60.8%)	119 (58.6%)	27 (73%)	0.070	-
Smoking history, n (%)	140 (58.3%)	116 (57.1%)	24 (64.9%)	0.245	0.987
Hospitalization within past 90 days, n (%)	51 (21.3%)	38 (18.7%)	13 (35.1%)	**0.025**	0.066
Recent antibiotic use, n (%)	93 (38.8%)	72 (35.5%)	21 (56.8%)	**0.013**	**0.019**
Any comorbidity, n (%)	207 (86.3%)	172 (84.7%)	35 (94.6%)	0.082	0.317
COPD, n (%)	71 (29.6%)	58 (28.6%)	13 (35.1%)	0.268	0.476
Diabetes mellitus, n (%)	57 (23.8%)	49 (24.1%)	8 (21.6%)	0.462	0.804
Hypertension, n (%)	94 (39.2%)	79 (38.9%)	15 (40.5%)	0.318	0.577
Coronary heart disease, n (%)	63 (26.3%)	55 (27.1%)	8 (21.6%)	0.495	0.593
Cerebrovascular disease, n (%)	59 (24.6%)	46 (22.7%)	13 (35.1%)	0.082	0.256
Malignancy, n (%)	30 (12.5%)	18 (8.9%)	12 (34.4%)	**<0.001**	**-**
PSI score, mean ± SD	113.15 ± 35.07	107.38 ± 31.50	143.84 ± 37.50	**<0.001**	**-**
CURB-65 ≥ 3, n (%)	33 (13.8%)	20 (9.9%)	13 (%35.1)	**<0.001**	**<0.001**
WBC (×10^9^/L)	11.81 [0.64–43.48]	11.82 [1.06–43.48]	11.80 [0.64–40.10]	0.366	0.206
Neutrophil (×10^9^/L)	9.975 [0.47–42.06]	9.90 [0.57–42.06]	10.17 [0.47–38.30]	0.204	0.154
Lymphocyte (×10^9^/L)	0.89 [0.04–6.60]	0.91 [0.04–6.60]	0.73 [0.14–2.67]	**0.013**	0.067
Monocyte (×10^9^/L)	0.57 [0–3.30]	0.57 [0.01–3.30]	0.50 [0–2.45]	0.550	0.619
Platelet (×10^9^/L)	238.50 [30–1275]	238 [30–697]	242 [109–1275]	0.685	0.354
SII (×10^3^)	2.61 [0–56.90]	2.57 [0–56.90]	3.01 [0.81–56.37]	**0.043**	0.063
SIRI	5.49 [0–83.75]	5.32 [0.06–83.75]	7.66 [0–64.88]	0.072	**0.046**
CRP (mg/L)	135 [3.11–417]	129 [4.41–417]	169.31 ± 100.69	0.157	0.094
Procalcitonin (ng/mL)	0.40 [0.01–100]	0.3 [0.01–100]	1.16 [0.03–70]	**0.014**	0.235

Data are expressed as mean ± SD or median [IQR] and n (%), SII = (Platelet × Neutrophil/Lymphocyte), SIRI = (Neutrophil × Monocyte/Lymphocyte). *p* < 0.05 was considered statistically significant. * Adjusted *p*-values were obtained from multivariable binary logistic regression models including age, sex, and malignancy as covariates. Bold *p*-values indicate statistical significance at *p* < 0.05.

**Table 2:** Correlations between baseline inflammatory biomarkers and clinical severity scores at admission.

	PSI Score	CURB-65	SII	SIRI	CRP	Procalcitonin
PSI Score	1.000	**0.636**	**0.199**	0.132	0.038	0.158
CURB-65		1.000	**0.216**	**0.183**	0.125	**0.245**
SII			1.000	**0.711**	**0.275**	**0.209**
SIRI				1.000	**0.347**	**0.278**
CRP					1.000	**0.451**
Procalcitonin						1.000

PSI, Pneumonia Severity Index; SII, systemic immune-inflammation index; SIRI, systemic inflammation response index; CRP, C-reactive protein. Bold correlation coefficients indicate statistical significance at *p* < 0.01.

**Table 3:** Univariate and bivariate binary logistic regression analyses of CURB-65, PSI, systemic inflammatory indices (SII and SIRI), and inflammatory biomarkers (CRP and procalcitonin) for predicting 30-day mortality.

Variable/Model	B	SE	Wald	OR (Exp B)	95% CI for OR	*p* Value
Model 1 CURB-65	1.058	0.263	16.125	2.879	1.718–4.825	<0.001
Model 2 PSI	0.035	0.007	28.233	1.036	1.022–1.049	<0.001
Model 3 SII	0.453	0.181	6.257	1.574	1.103–2.245	0.012
Model 4 SIRI	0.367	0.170	4.685	1.444	1.035–2.013	0.030
Model 5 CRP	0.003	0.002	2.421	1.003	0.999–1.007	0.120
Model 6 Procalcitonin	0.015	0.011	1.919	1.015	0.994–1.038	0.166
Model 7 CURB-65 SII	1.024 0.000	0.266 0.000	14.846 3.384	2.783 1.000	1.654–4.685 1.000–1.000	<0.001 0.066
Model-8 CURB-65 SIRI	1.041 0.025	0.268 0.013	15.084 3.773	2.831 1.026	1.674–4.786 1.000–1.052	<0.001 0.052
Model-9 PSI SII	0.034 0.000	0.007 0.000	26.242 2.066	1.035 1.000	1.021–1.048 1.000–1.000	<0.001 0.151
Model-10 PSI SIRI	0.035 0.025	0.007 0.014	26.855 3.415	1.035 1.026	1.022–1.049 0.998–1.054	<0.001 0.065

B, regression coefficient; SE, standard error; OR (Exp B), odds ratio; CI, confidence interval; PSI, pneumonia severity index; SII, systemic immune-inflammation index; SIRI, systemic inflammation response index; CRP, C-reactive protein. SII and SIRI were log-transformed before inclusion in the models.

**Table 4:** Receiver operating characteristic (ROC) analysis of systemic inflammatory indices, inflammatory biomarkers (CRP and procalcitonin), and clinical scores with and without SII/SIRI for predicting 30-day mortality.

	AUC (95% CI)	SE	*p* (vs. 0.5)	ΔAUC (vs. Base Model)	95% CI for ΔAUC	*p* (DeLong)	Optimal Cut-Off	Sensitivity, %	Specificity, %
CRP	0.569 (0.467–0.671)	0.052	0.187	-	-	-	-	-	-
PCT	0.627 (0.531–0.723)	0.049	0.010				>1.16	51.4	72.7
SII	0.605 (0.540–0.667)	0.053	0.047	-	-	-	>8.53	43.2	76.9
SIRI	0.609 (0.544–0.672)	0.054	0.042	-	-	-	>1.68	69.4	50.3
CURB-65	0.684 (0.621–0.742)	0.043	<0.001	-	-	-	>2	35.1	90.2
CURB-65 + SII	0.723 (0.662–0.779)	0.046	<0.001	0.039	−0.007–0.085	0.098			
CURB-65 + SIRI	0.727 (0.665–0.782)	0.048	<0.001	0.043	−0.007–0.091	0.091			
PSI	0.768 (0.709–0.820)	0.046	<0.001	-	-	-	>140	62.2	84.7
PSI + SII	0.774 (0.715–0.825)	0.045	<0.001	0.006	−0.012–0.023	0.533			
PSI + SIRI	0.776 (0.718–0.827)	0.046	<0.001	0.008	−0.017–0.033	0.543			

AUC, area under the ROC curve; CI, confidence interval; SE, standard error; CRP, C-reactive protein; PCT, procalcitonin; PSI, pneumonia severity index; SII, systemic immune-inflammation index; SIRI, systemic inflammation response index; “–” indicates that the value was not calculated (not applicable). Pairwise AUC differences were assessed using the DeLong test. CURB-65 and PSI denote baselineclinical models. SII and SIRI were log-transformed before inclusion in the models.

## Data Availability

The data presented in this study are available upon reasonable request from the corresponding author. The data are not publicly available due to ethical restrictions.
